# Constructing the Logical Regression Model to Predict the Target of Jianpi Jiedu Decoction in the Treatment of Hepatocellular Carcinoma

**DOI:** 10.1155/2020/8859558

**Published:** 2020-12-28

**Authors:** Rongjie Zhang, Yan Chen, Ge Zhou, Baoguo Sun, Yue Li, Zexiong Chen

**Affiliations:** ^1^Department of Traditional Chinese Medicine, The First Affiliated Hospital of Sun Yat-Sen University, Guangzhou 510000, China; ^2^Department of Traditional Chinese Medicine, The Third Affiliated Hospital of Sun Yat-Sen University, Guangzhou 510630, China

## Abstract

**Objectives:**

The purpose of this study was to identify the molecular mechanism and prognosis-related genes of Jianpi Jiedu decoction in the treatment of hepatocellular carcinoma.

**Methods:**

The gene expression data of hepatocellular carcinoma samples and normal tissue samples were downloaded from TCGA database, and the potential targets of drug composition of Jianpi Jiedu decoction were obtained from TCMSP database. The genes were screened out in order to obtain the expression of these target genes in patients with hepatocellular carcinoma. The differential expression of target genes was analyzed by R software, and the genes related to prognosis were screened by univariate Cox regression analysis. Then, the LASSO model was constructed for risk assessment and survival analysis between different risk groups. At the same time, independent prognostic analysis, GSEA analysis, and prognostic analysis of single gene in patients with hepatocellular carcinoma were performed.

**Results:**

174 compounds of traditional Chinese medicine were screened by TCMSP database, corresponding to 122 potential targets. 39 upregulated genes and 9 downregulated genes were screened out. A total of 20 candidate prognostic related genes were screened out by univariate Cox analysis, of which 12 prognostic genes were involved in the construction of the LASSO regression model. There was a significant difference in survival time between the high-risk group and low-risk group (*p* < 0.05). Among the genes related to prognosis, the expression levels of CCNB1, NQO1, NUF2, and CHEK1 were high in tumor tissues (*p* < 0.05). Survival analysis showed that the high expression levels of these four genes were significantly correlated with poor prognosis of HCC (*p* < 0.05). GSEA analysis showed that the main KEGG enrichment pathways were lysine degradation, folate carbon pool, citrate cycle, and transcription factors.

**Conclusions:**

In the study, we found that therapy target genes of Jianpi Jiedu decoction were mainly involved in metabolism and apoptosis in hepatocellular carcinoma, and there was a close relationship between the prognosis of hepatocellular carcinoma and the genes of CCNB1, NQO1, NUF2, and CHEK1.

## 1. Introduction

Primary liver cancer (PLC) is a malignant tumor with malignant transformation of hepatocytes, intrahepatic bile duct cells, or a mixture of the two. Hepatocellular carcinoma (HCC) ranks third among cancer-related deaths [1, 2]. Epidemiology shows that HCC has become the second leading cause of death of malignant tumors in the world. There are more than 500,000 HCC patients in the world every year, and more than half of them are in China [3]. And HCC is the third leading cause of death among fatal tumors in China [4]. The onset of HCC is hidden, but the disease develops rapidly. Multidisciplinary comprehensive treatment is an important treatment for HCC patients without the opportunity of surgical resection. The treatment of HCC with traditional Chinese medicine plays an important role in reducing side effects, delaying the progression, improving the quality of life, and prolonging the survival time [5]. In addition, studies have confirmed that many traditional Chinese medicines have good antitumor activity [6–8].

Jianpi Jiedu decoction (JJD) is an empirical prescription for HCC which was founded by the Department of Traditional Chinese Medicine of the First Affiliated Hospital of Sun Yat-Sen University. The main components of JJD contain Codonopsis Radix 30 g, Poria 15 g, *Atractylodes macrocephala* 15 g, licorice 6 g, *Bupleurum* 12 g, Rhizoma Zedoariae 15 g, and *Scutellaria barbata* 30 g. Previous studies showed that JJD could improve the food intake of the mice with HCC cachexia effectively, maintain the bodyweight, and prolong the survival time of tumor-bearing mice by regulating the expression of PTEN and FAK [9–11]. Clinical studies also showed that JJD could promote the recovery of liver function of HCC patients, improve the postoperative adverse reactions, and improve the quality of life of HCC patients [12].

There are many studies on JJD, but the molecular mechanism of JJD in the treatment of HCC has not been fully elucidated. In this study, in order to explore the mechanism of JJD in the treatment of HCC, the target genes of JJD in the treatment of HCC were screened out by combining TCMSP database and TCGA database, a logical regression model was constructed, the core genes related to prognostic risk were screened out, the relationship between their expression and prognosis was analyzed, and the functions of these genes were also analyzed by GSEA.

## 2. Materials and Methods

### 2.1. Gene Data Acquisition of HCC

**Figure 1:**
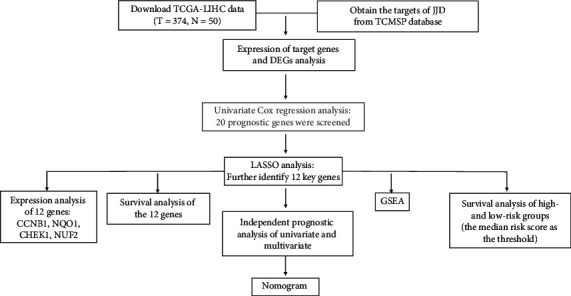
The flowchart of the study.

The study followed the flowchart (Figure 1). The Cancer Genome Atlas (TCGA) database was used to obtain the data of genes expression and clinical information of LIHC (liver hepatocellular carcinoma). The research was in line with the publication guidelines provided by TCGA. And the data of genes expression and corresponding clinical characteristic of 374 HCC sample files and 50 normal sample files were obtained from TCGA database.

### 2.2. Acquisition of Drug Targets of JJD

The herbs of JJD include Codonopsis Radix, Poria, *Atractylodes macrocephala*, licorice, *Bupleurum*, Rhizoma Zedoariae, and *Scutellaria barbata*. The related chemical components of these herbs were obtained by TCMSP database [12] (Traditional Chinese Medicine Systems Pharmacology Database and Analysis Platform, http://tcmspw.com/tcmsp.php). The criteria for screening effective compounds were oral bioavailability (OB ≥ 30%) and drug-like drugs (DL ≥ 0.18), and the corresponding target genes of these effective compounds were obtained. With the help of UniProt database, the species was limited to *Homo sapiens*, and the target was converted to the corresponding gene symbol.

### 2.3. Differential Expression Genes (DEGs) Analysis

The target genes of JJD were mapped to LIHC gene expression data to obtain the expression of target genes. The limma package of R software was used to correct the data, and false discovery rate (FDR) < 0.05 and |log2 fold change (log2FC)| ≥ 1.0 were set as the cutoffs to screen for DEGs.

### 2.4. Construction of the LASSO Model and Independent Prognostic Analysis

Using the survival package of R language, univariate Cox regression analysis was performed to evaluate the correlation between target genes and survival risk in patients with LIHC. Furthermore, the least absolute shrinkage and selection operator (LASSO) model was constructed, and the best *λ* value was selected to select the optimal prediction characteristics of risk factors. According to the results of LASSO regression analysis, the risk score curve was drawn, and the relationships between the survival status and risk score and the gene expression and risk score were analyzed. Taking the median risk coefficient as the limit, the patient sample data were divided into the high-risk group and low-risk group, and the survival analysis was carried out to analyze the relationship between the risk score and patient survival time. According to the clinical characteristics of the American Joint Committee on Cancer (AJCC) stage, T stage, M stage, N stage, and risk factors, the nomogram was constructed by Cox analysis to evaluate the 1-year, 3-year, and 5-year survival rate of patients.

### 2.5. Identification of Key Genes Related to Prognosis

GEPIA2 [13] (gene expression profiling interactive analysis, http://gepia2.cancer-pku.cn) is a web server and an online database. GEPIA2 database was used to analyze the expression of prognostic genes included in the LASSO model in tumor tissues and normal tissues. And the analysis condition was set to logFC = 1, cutoff = 0.05, match TCGA normal, and GTEx data. Finally, single gene survival analysis was carried out.

### 2.6. GSEA (Gene Set Enrichment Analysis)

Gene data from the LASSO model was used for GSEA analysis. The number of permutations was set to 1000. Using GSEA 4.0.3 software, the biological processes (BP) and KEGG (Kyoto Encyclopedia of Genes and Genomes) pathways of the gene were analyzed, and the main biological pathways involved in genes associated with high-risk groups were studied.

## 3. Results

### 3.1. Identification of DEGs

A total of 18752 genes associated with HCC were screened out of the raw counts of RNA-sequencing data from the TCGA dataset. JJD contains Codonopsis Radix, Poria, *Atractylodes macrocephala*, licorice, *Bupleurum*, Rhizoma Zedoariae, and *Scutellaria barbata*, a total of 7 herbs. 174 active compounds of traditional Chinese medicine were screened by TCMSP (Table 1). By mapping with the data of gene expression of HCC patients, a total of 122 genes target to HCC were identified from the 174 active compounds of traditional Chinese medicine (Table 1). According to the differential analysis of gene expression, a total of 48 DEGs were screened out among the 122 genes, including 39 upregulated genes and 9 downregulated genes (Table 2).

### 3.2. Screening and Verification of Prognosis-Related DEGs

Analyzing the above 48 DEGs further, a total of 20 candidate genes related to prognostic risk were screened out by univariate Cox analysis (Table 3). And the high-risk genes contained CASP3, CASP8, CHEK1, AHSA1, ACACA, BIRC5, CCNB1, NQO1, HSF1, RASSF1, ERBB3, NPEPPS, PCNA, NUF2, AKR1C3, ABCC1, and AKR1C1 (HR > 1, *p* < 0.05). Low-risk genes included ESR1, SLC2A4, and ABAT (HR < 1, *p* < 0.05).

**Figure 2:**
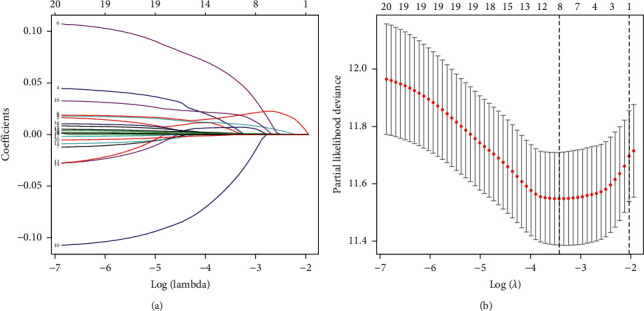
LASSO regression model was performed to further screen prognostic genes. (a) Lasso coefficient profiles with 20 characteristics. The coefficient profile was generated by comparing with the log (*λ*) sequence. 12 predictors of nonzero coefficients were obtained after selecting the optimal *λ* value (also known as the minimum *λ* value = 0.0324). (b) The selection of the optimal parameter (*λ*) in the LASSO model was cross-verified for 1000 times by the minimum standard. Draw a point vertical line at the optimal value by using 1 standard error (SE) of the minimum standard the minimum standard (1-SE standard). When choosing the optimal log (*λ*) value (the first vertical line), the model included 12 nonzero prediction values.

**Figure 3:**
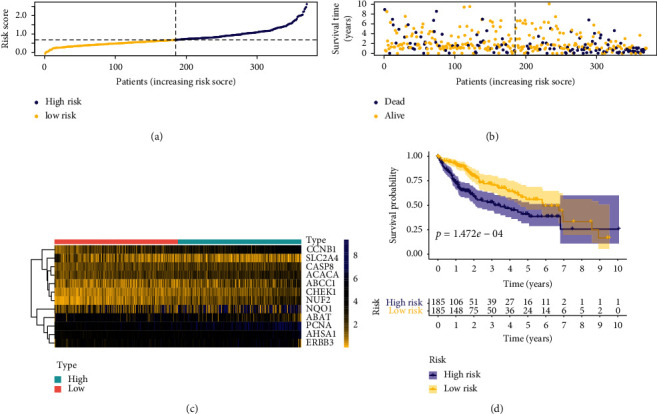
(a) Risk score distribution map of HCC patients. The LASSO regression model was constructed; yellow represents the low-risk group and blue represents the high-risk group. (b) Distribution map of survival status of HCC patients. The *x-*axis is the risk score, the *y-*axis is the patient's survival time, the yellow indicates that the patient's status was alive, and the blue color indicates that the patient's status was dead. (c) The heat maps of 12 genes involved in the construction of the model. Yellow indicates low gene expression, black implies moderate gene expression, and blue implies high gene expression. From left to right, gene expression changed with the increase of the risk score. (d) Survival curve of HCC patients. With the median risk score as the threshold, the patients were divided into two groups. Yellow implies the low-risk group, while blue implies the high-risk group.

To further identify the 20 candidate prognostic genes which were correlated significantly with the prognosis of HCC patients, LASSO regression with 1000 times cross-validation was performed to get the optimal lambda value that came from the minimum partial likelihood deviance (lambda = 0.0324). Finally, 12 genes were screened out from the 20 candidate prognostic genes (Figures 2(a) and 2(b)). The 12 genes were CASP8, CHEK1, AHSA1, ACACA, CCNB1, NQO1, ERBB3, PCNA, NUF2, ABCC1, ABAT and SLC2A4, respectively (Table 4). The expression levels of ABAT and SLC2A4 were negatively correlated with risk score, while the expression of other genes was positively correlated with risk score (Table 4). The heatmap of 12 prognostic genes is presented in Figure 3(c). The risk score of every HCC patient was calculated according to the risk coefficient values in Table 4. All HCC patients were ranked according to their risk score from low to high, and they were divided into the low-risk group and high-risk group from left to right (Figure 2(a)). Survival analysis was carried out for patients in the low-risk group and high-risk group (Figure 2(b)). Through the construction of the LASSO regression model, the risk score of HCC patients was ranked from low to high. According to the median risk score, HCC patients were divided into the low-risk group and high-risk group (Figure 3(a)). The distribution map of survival status of HCC patients showed that the mortality rate of patients increased with the increase of risk scores (Figure 3(b)). Survival curve of HCC patients displayed that the overall survival time of the high-risk group was significantly worse than that of the low-risk group (Figure 3(d), *p* < 0.05).

### 3.3. Establishment and Estimation of the 12-Gene Prognostic Signature

**Figure 4:**
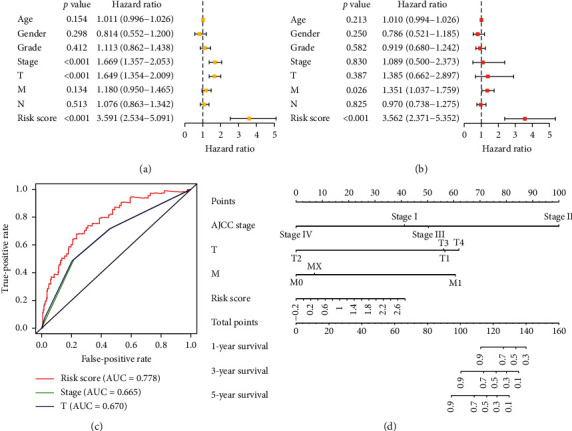
Assessment of the 12-gene prognostic signature and a compound nomogram establishment. (a) Univariate independent prognostic analysis chart, we analyzed age, gender, grade, stages T, M, and N, and risk score. And stage T was the high risk independent prognostic factor (*p* < 0.05). (b) Multivariate independent prognostic analysis showed that M and risk score were high risk factors (*p* < 0.05). (c) ROC curve showed that the risk score (AUC = 0.776) had better prediction performance than stage (AUC = 0.665), T (AUC = 0.670), and other factors. (d) Nomogram. According to AJCC stage, T, M, and N and risk factors, the model was constructed by Cox analysis, and the 1-year, 3-year, and 5-year survival rate of patients with HCC was evaluated according to the total score of clinical characteristics.

In the results of independent prognostic analysis, univariate Cox regression analysis showed that AJCC-stage, T stage, and risk score were correlated with the survival rate of HCC patients (*p* < 0.05, Figure 4(a)). Multivariate Cox regression analysis showed that the M stage and risk score could be used as specific prognostic indicators of HCC (*p* < 0.05, Figure 4(b)). ROC curve showed that the AUC (area under curve) of the risk score was higher than AJCC-stage and T staging, that is, the risk score showed better predictive performance (Figure 4(c)). The clinical pathological parameters of HCC patients in this study are shown in Table 5. In order to establish a more reliable predictive method for clinical practice, we constructed a compound nomogram integrating the risk score, AJCC stage, T stage, and M stage to predict 1-year, 3-year, and 5-year survival rate of HCC patients (Figure 4(d)).

### 3.4. Identification of Key Genes Related to Prognosis

**Figure 5:**
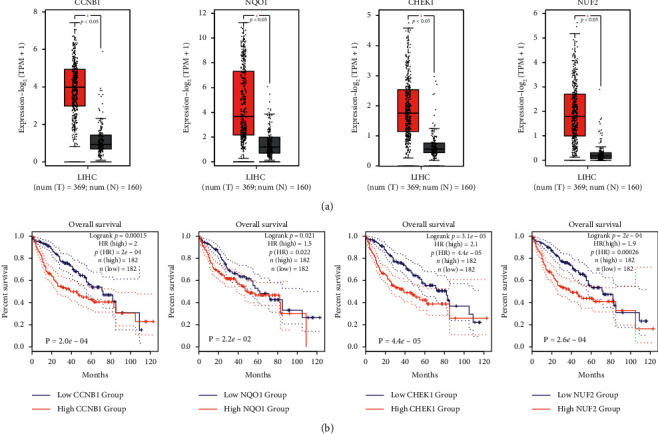
Identification of key genes related to prognosis. (a) Expression of CCNB1, NQO1, CHEK1, and NUF2 genes in the tumor group and normal group. Red indicates the tumor group, and grey indicates the normal group (^*∗*^*p* < 0.05). (b) The relationship between CCNB1, NQO1, CHEK1, and NUF2 gene expression and survival time of patients. Red implies the group with high gene expression. Blue implies the group of low gene expression (^*∗*^*p* < 0.05).

The study showed that the expression levels of CCNB1, NQO1, CHEK1, and NUF2 in the HCC group were significantly higher than the normal sample group through further analysis of the 12-gene from GEPIA2 database (*p* < 0.05, Figure 5(a)). And the prognosis of patients with high expression of CCNB1, NQO1, CHEK1, and NUF2 genes was worse than the patients with low expression (*p* < 0.05, Figure 5(b)), so the four genes might be related to the poor prognosis of the patients.

### 3.5. Identification of Biological Pathways of DEGs

**Figure 6:**
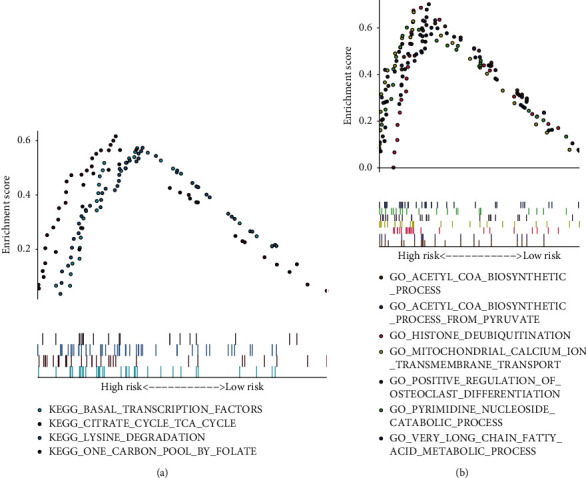
GSEA analysis: KEGG pathways (upper side) and biological processes (lower side). KEGG pathway mainly included lysine degradation, folate carbon pool, citrate cycle, and transcription factor. Biological processes included mitochondrial calcium ion transmembrane transport, acetyl-CoA biosynthesis, long-chain fatty acid metabolism, nucleoside catabolism, pyruvate acetyl-CoA biosynthesis, dehistone de-disproportionation, and so on.

KEGG pathway analysis showed that the main pathways involved in genes in the high-risk group were lysine degradation, folate carbon pool, citrate cycle, and basic transcription factor processes. BP included mitochondrial calcium transport, acetyl-CoA biosynthesis, long-chain fatty acid metabolism, nucleoside catabolism, pyruvate acetyl-CoA biosynthesis, dehistone disproportionation, and so on (Table 6 and Figure 6). Therefore, the mechanism of JJD in the treatment of HCC might be related with these pathways.

## 4. Discussion

HCC is one of the common types of cancer with the hidden onset, rapid progress, and easy metastasis. In recent years, HCC becomes a worldwide social and clinical problem because of its rising prevalence. At present, the common treatments for HCC include surgery, transcatheter arterial chemoembolization, radiofrequency ablation, molecular targeted therapy, immunotherapy, and so on [14]. However, morbidity and cancer-specific mortality remain at a high level, although comprehensive measures have shown effectiveness in preventing HCC. In addition, most of the patients with HCC are in the advanced stage with an unsatisfactory prognosis [15].

As an adjuvant therapy, traditional Chinese medicine can achieve the effect of increasing efficiency and reducing toxicity. The combination of Chinese and Western medicine can improve the efficacy of Western medicine, delay the development, and improve the survival rate of HCC. For example, TACE combined with traditional Chinese medicine can improve the prognosis and prolong the survival time of patients with unresectable HCC [5]. Surgery combined with traditional Chinese medicine can reduce the recurrence rate and prolong the survival time for HCC patients with small tumor or a single tumor with the diameter of less than 5 cm [16]. JJD contains 7 herbs. *Bupleurum* can soothe the liver and relieve depression; *Scutellaria barbata* and Rhizoma Zedoariae can clear heat and detoxify, remove qi, and disperse knots; and Codonopsis Radix, *Atractylodes macrocephala*, Poria, and licorice can replenish qi and invigorate the spleen; thus, satisfactory results have been obtained in the treatment of liver cancer.

In this study, the logical regression model was established by using the methods of network pharmacology and bioinformatics, mainly to study the target of JJD and identify the prognostic genes of HCC.

The study showed that the functions of these genes were mainly related to the metabolism of a variety of substances according to the functional analysis of the genes based on the regression model. It was consistent with the previous studies that JJD could downregulate ABCC2 and upregulate OATP1B2 in liver cancer tissue. JJD also could promote food intake, reduce diarrhea, and improve metabolism of rats with HCC [9]. Studies have proved that specific metabolic dependence in cancer was also the basis of effective treatment, including IDH1 inhibitors, folic acid and thymidine metabolism, lipid metabolism, and so on [17]. At present, there are several approved drugs for systematic treatment of HCC, such as sorafenib, lenvatinib, regorafenib, cabozantinib, and nivolumab [18]. However, none of these treatments can solve the changes of metabolic function of HCC cells. So, the further research on metabolism of HCC is necessary.

In addition, the study showed that the genes of CCNB1, NQO1, CHEK1, and NUF2 were closely related to the prognosis of HCC, which might also be the key target of JJD in the treatment of HCC.

NQO1 mainly encodes an intracellular detoxification enzyme that catalyzes quinone to hydroquinone and maintains intracellular redox homeostasis with NADH and NADPH as electron donors. Studies have shown that overexpressed NQO1 can enhance the apoptosis escape and play an important role in maintaining the proliferation of hepatocellular carcinoma cells. High expression of NQO1 can inhibit 26S proteasome degradation mediated by ubiquitin to enhance the stability of SIRT6 protein, lead to the increase of AKT phosphorylation and activity, further affect the stability of antiapoptotic protein XIAP, and enhance the apoptotic escape of hepatocellular carcinoma cells [19]. At the same time, NQO1 can regulate Akt and c-Myc through MAPK/ERK and PI3K/Akt signal pathways to regulate glucose and glutamine metabolism, which is closely related to the uncontrolled proliferation of cancer cells [20].

NUF2 is an important part of NDC80 kinetoplast complex, which is essential for kinetochore-microtubule attachment and chromosome segregation. Genomic instability and aneuploidy have been considered to be the characteristics of cancer [21]. As the core components of the NDC80 kinetoplast complex, NUF2 has been reported to be associated with a variety of human tumorigenesis. In terms of liver cancer, some experiments have shown that the expression of NUF2 in human liver cancer tissues is significantly higher than that in adjacent normal tissues, and there is also a high expression in liver cancer cell lines. High expression of NUF2 can promote the growth and inhibit the apoptosis of liver cancer cells. On the contrary, the absence of NUF2 significantly inhibited cell proliferation and colony formation in vitro and significantly hindered the growth of transplanted tumors in vivo. Moreover, NUF2 silencing can induce cell cycle arrest and trigger apoptosis [22]. Through the genome-wide expression microarray analysis of recurrent HCC, it is found that NUF2 complicated with liver cirrhosis can be used as a promising prognostic biomarker of early HCC recurrence [23].

CHKE1, known as CHK1, is a necessary kinase to maintain genomic stability; its function was mainly related to DNA damage activation-checkpoint. In particular, ataxia telangiectasia and Rad3-related (ATR)-CHK1 signal cascades are the main factors of replication stress response. The signal cascade has a variety of functions, including regulating the initiation of DNA replication, stably stagnating replication forks, and delaying the entry of mitosis by preventing the overactivation of cyclin-dependent kinases (CDK) 1 and 2 [24]. Some studies have suggested that because of the key role of CHKE1 in regulating checkpoints induced by DNA damage, and cancer cells usually lack normal G1 checkpoint control, and they may be more dependent on S and G2 checkpoints than normal cells [25]. Standard chemotherapy or radiotherapy can be combined with drugs that inhibit CHK1 kinase to inhibit S and G2 checkpoints. Cancer cells may be particularly sensitive to this treatment. Therefore, CHK1 has been considered as a potential target for cancer therapy [26]. It is worth noting that some studies have suggested that CHK1 is necessary in the normal S phase to avoid the abnormal increase of DNA replication, so as to protect DNA from being destroyed, which is an important kinase. This should be noted when considering the use of drugs that inhibit the kinase for cancer treatment [27].

The protein encoded by CCNB1 is a regulatory protein involved in mitosis, which is necessary for the correct control of the transition phase of cell cycle G2/M. Studies proved that overexpressed CCNB1 could block the p53 signal pathway and regulate apoptosis, invasion, and cell cycle of hepatocellular carcinoma cells by inhibiting the expression of p53 and p21 proteins. On the contrary, silencing CCNB1 gene could promote the expression of p53 and p21 proteins in hepatoma cells, promote apoptosis of HepG2 and Huh-7 cells, inhibit cell invasion, and block cells in the G0/G1 phase [28]. In addition, some studies have suggested that CCNB1 can be used as an independent predictor of HCC recurrence (hazard ratio (HR), 4.762; *p*=0.002), which is significantly related to the overall survival rate of HBV-related HCC recurrence [29].

In a word, as a compound prescription of traditional Chinese medicine, the prescription of traditional Chinese medicine has multitargets and multipathways in the treatment of diseases because of the complex composition. This study showed that the mechanism of JJD in the treatment of HCC might be mainly through the intervention of HCC metabolism and the regulation of apoptosis according to the analysis of network pharmacology and bioinformatics. And the genes of CCNB1, NQO1, CHEK1, and NUF2 may be the key targets of JJD in the treatment of HCC. But the further study of the conclusion is needed.

## Figures and Tables

**Table 1:** Composition of JPJDD and Mol ID of traditional Chinese medicine compound and target symbol.

Composition of JPJDD	Mol ID	Symbol
Poria cocos (Schw.) Wolf	MOL000282, MOL000291, MOL000296, MOL000292, MOL000290, MOL000279, MOL000275, MOL000273, MOL000289, MOL000276, MOL000287, MOL000283, MOL000280, MOL000285, MOL000300	PGR, NCOA2, CHRM3, CHRM1, CHRM2, GABRA1, GRIA2, ADH1B, PTGS1, NR3C2

Atractylodes macrocephala Koidz	MOL000072, MOL000033, MOL000028, MOL000049, MOL000021, MOL000020, MOL000022,	GABRA1, NCOA2, NCOA1, PGR, CHRM3, CHRM1, AR, ACHE, ADRA1A, CHRM2

Rhizoma Zedoariae	MOL000910, MOL00091	PRSS1, PGR, NCOA2, CHRM3, CHRM1, CHRM2, GABRA1, GRIA2, ADH1B, PTGS1

Scutellariae barbata Herba	MOL012269, MOL001973, MOL012252, MOL000449, MOL001755, MOL002776, MOL000358, MOL000359, MOL005869, MOL012270, MOL012254, MOL000953, MOL012266, MOL000351, MOL012248, MOL002915, MOL000098, MOL012245, MOL001735, MOL012246, MOL008206, MOL012250, MOL000006, MOL005190, MOL002719, MOL000173, MOL001040, MOL002714, MOL012251,	PGR, NR3C2, NCOA2, NCOA1, PTGS1, AKR1B1, PLAU, CTRB1, CHRM3, CHRM1, ADRA1A, CHRM2, GABRA1, CHRM4, CHRNA2, BCL2, CASP9, CASP3, CASP8, PRKCA, PON1, AR, F7, ESR2, PRSS1, ESR1, PPARG, GSK3B, CHEK1, ACHE, RELA, EGFR, VEGFA, CCND1, FOS, EIF6, RB1, IL6, AHSA1, TP63, ELK1, NFKBIA, POR, RAF1, HIF1A, RUNX1T1, ERBB2, ACACA, CYP3A4, CAV1, MYC, CYP1A1, ICAM1, SELE, VCAM1, PTGER3, BIRC5, DUOX2, NOS3, HSPB1, MGAM, CYP1B1, CCNB1, ALOX5, GSTP1, NFE2L2, NQO1, PARP1, AHR, PSMD3, SLC2A4, COL3A1, DCAF5, NR1I3, CHEK2, HSF1, CRP, RUNX2, RASSF1, CTSD, IGFBP3, IGF2, IRF1, ERBB3, DIO1, NPEPPS, HK2, RASA1, GSTM1, GSTM2, RHO, MDM2, APP, PCNA, CASP7, MCL1, TYR, NUF2, TEP1, NR3C1, FOSL1, CYCS, NOX5, APOD

Radix Bupleuri	MOL001645, MOL004644, MOL000422, MOL004648, MOL000098, MOL004628, MOL000490, MOL000354, MOL004609, MOL004624, MOL004598, MOL004702, MOL013187, MOL004653, MOL002776, MOL004718, MOL000449,	PTGS1, NCOA2, AR, PPARG, PRSS1, PGR, CHRM1, ACHE, CHRM2, GABRA1, F7, RELA, IKBKB, BCL2, AHSA1, CASP3, MAPK8, CYP3A4, CYP1A1, ICAM1, SELE, VCAM1, CYP1B1, ALOX5, GSTP1, AHR, PSMD3, SLC2A4, NR1I3, DIO1, GSTM1, GSTM2, AKR1C3, AKR1B1, EGFR, VEGFA, CCND1, FOS, EIF6, CASP9, PLAU, RB1, IL6, TP63, ELK1, NFKBIA, POR, CASP8, RAF1, PRKCA, HIF1A, RUNX1T1, ERBB2, ACACA, CAV1, MYC, PTGER3, BIRC5, DUOX2, NOS3, HSPB1, MGAM, CCNB1, NFE2L2, NQO1, PARP1, COL3A1, DCAF5, CHEK2, HSF1, CRP, RUNX2, RASSF1, CTSD, IGFBP3, IGF2, IRF1, ERBB3, PON1, NPEPPS, HK2, RASA1, ESR2, GSK3B, ESR1, CHEK1, NCOA1, GRIA2, OLR1, NR3C2, CTRB1, CHRM3, ADRA1A

Codonopsis Radix	MOL003896, MOL007514, MOL008400, MOL000006, MOL002140, MOL008393, MOL005321, MOL002879, MOL008406, MOL007059, MOL004492, MOL008411, MOL006774, MOL001006, MOL004355, MOL003036, MOL000449, MOL008407, MOL006554, MOL008397, MOL008391	PTGS1, CHRM3, CHRM1, ESR1, AR, PPARG, ACHE, ESR2, GABRA1, GSK3B, CHEK1, PRSS1, NCOA1, CHRM5, NCOA2, APP, RELA, EGFR, VEGFA, CCND1, CASP9, RB1, IL6, CASP3, TP63, NFKBIA, MDM2, PCNA, ERBB2, CASP7, ICAM1, MCL1, BIRC5, CCNB1, TYR, GSTP1, SLC2A4, NUF2, TUBB1, ADRA1A, PGR, NR3C2, AKR1B1, PLAU, CTRB1, CHRM2, NR3C1,

Licorice	MOL001792, MOL002844, MOL004941, MOL004835, MOL004841, MOL004985, MOL004996, MOL003896, MOL004957, MOL004328, MOL000392, MOL000500, MOL000422, MOL000417, MOL004991, MOL004860, MOL004990, MOL000098, MOL000497, MOL000239, MOL005016, MOL004898, MOL000354, MOL004910, MOL004945, MOL004848, MOL004980, MOL004961, MOL002565, MOL004829, MOL004815, MOL004828, MOL004907, MOL004882, MOL004915, MOL003656, MOL005020, MOL004838, MOL005000, MOL004811, MOL004856, MOL004993, MOL004864, MOL004935, MOL004989, MOL004866, MOL004863, MOL004814, MOL004883, MOL004949, MOL004913, MOL004849, MOL004911, MOL004808, MOL004833, MOL004857, MOL004879, MOL004908, MOL004855, MOL005012, MOL004912, MOL004978, MOL004820, MOL004914, MOL004885, MOL001484, MOL004810, MOL004905, MOL004884, MOL004827, MOL004806, MOL004966, MOL004974, MOL005003, MOL005017, MOL005007, MOL005008, MOL004824, MOL004959, MOL004904, MOL002311, MOL005013, MOL004805, MOL004891, MOL004903, MOL000359, MOL005001, MOL000211, MOL004917, MOL004948, MOL005018, MOL004988, MOL004924	PTGS1, ESR1, GABRA1, CHRM1, AR, PPARG, ESR2, GSK3B, CHEK1, NCOA1, NCOA2, CHRM3, ACHE, PRSS1, CHRM5, RELA, BCL2, CASP3, FASN, LDLR, MTTP, APOB, GSTP1, SREBF1, ABCC1, AKR1C1, ABAT, ADRA1A, CHRM4, PGR, CHRM2, F7, IKBKB, AHSA1, MAPK8, CYP3A4, CYP1A1, ICAM1, SELE, VCAM1, CYP1B1, ALOX5, AHR, PSMD3, SLC2A4, NR1I3, DIO1, GSTM1, GSTM2, AKR1C3, AKR1B1, EGFR, VEGFA, CCND1, FOS, EIF6, CASP9, PLAU, RB1, IL6, TP63, ELK1, NFKBIA, POR, CASP8, RAF1, PRKCA, HIF1A, RUNX1T1, ERBB2, ACACA, CAV1, MYC, PTGER3, BIRC5, DUOX2, NOS3, HSPB1, MGAM, CCNB1, NFE2L2, NQO1, PARP1, COL3A1, DCAF5, CHEK2, HSF1, CRP, RUNX2, RASSF1, CTSD, IGFBP3, IGF2, IRF1, ERBB3, PON1, NPEPPS, HK2, RASA1, GRIA2, OLR1, MAPK10, RXRB, HTR3A, NR3C2

**Table 2:** Expression of JJD corresponding target in HCC.

DEGs	Symbol
Upregulated genes	HTR3A, NQO1, NUF2, BIRC5, RHO, CCNB1, CHEK1, CHRM4, HSPB1, AKR1C3, CYP1B1, CHRM3, PLAU, TP63, APOD, PTGER3, PCNA, ABCC1, FASN, CHEK2, ACACA, PRKCA, HSF1, ERBB3, ACHE, PARP1, RASSF1, AKR1C1, PPARG, CHRM5, RXRB, NPEPPS, CAV1, CASP8, AHSA1, SLC2A4, GSTM2, CASP3, CYCS
Downregulated genes	ABAT, CYP3A4, ADH1B, IGFBP3, IL6, ESR1, FOS, ADRA1A, CHRM2

**Table 3:** Results of univariate Cox analysis.

ID	HR	HR.95L	HR.95H	*P* value
CASP3	1.047	1.004	1.091	2.99*E* − 02
CASP8	1.19	1.067	1.328	1.79*E* − 03
ESR1	0.753	0.567	0.999	4.92*E* − 02
CHEK1	1.384	1.211	1.581	1.85*E* − 06
AHSA1	1.035	1.021	1.048	5.67*E* − 07
ACACA	1.123	1.054	1.196	3.39*E* − 04
BIRC5	1.027	1.013	1.04	7.03*E* − 05
CCNB1	1.044	1.029	1.059	2.42*E* − 09
NQO1	1.002	1.001	1.003	2.81*E* − 04
SLC2A4	0.872	0.774	0.982	2.44*E* − 02
HSF1	1.019	1.004	1.035	1.36*E* − 02
RASSF1	1.115	1.045	1.19	9.79*E* − 04
ERBB3	1.009	1.001	1.017	2.97*E* − 02
NPEPPS	1.061	1.012	1.113	1.40*E* − 02
PCNA	1.011	1.006	1.016	6.58*E* − 06
NUF2	1.12	1.068	1.175	2.90*E* − 06
AKR1C3	1.003	1.001	1.005	9.56*E* − 04
ABCC1	1.072	1.035	1.111	1.12*E* − 04
AKR1C1	1.002	1.001	1.004	7.23*E* − 03
ABAT	0.987	0.978	0.996	5.38*E* − 03

Note: univariate Cox analysis was performed. HR.95L−HR.96H was the 95% confidence interval of HR.

**Table 4:** Results of LASSO regression analysis.

Gene	Coefficient
CASP8	0.003428
CHEK1	0.006758
AHSA1	0.010426
ACACA	0.054369
CCNB1	0.01794
NQO1	0.001041
SLC2A4	−0.04431
ERBB3	0.00104
PCNA	0.000839
NUF2	0.006888
ABCC1	0.019659
ABAT	−0.00023

Note: coefficient was the risk coefficient of gene.

**Table 5:** Clinical pathological parameters of patients with HCC in this research.

Clinical characteristic	*N*	%
Age (years)		
≤30	15	4.0
30 ≤ *y* ≤ 60	165	44.2
>80	193	51.8
Gender		
Male	254	68.0
Female	120	32.0
T classification		
T1	185	49.6
T2	94	25.2
T3	81	21.7
T4	13	3.5
N classification		
N0	257	68.9
N1	4	1.1
Nx	112	30.0
M classification		
M0	271	72.4
M1	4	1.1
Mx	99	24.5
AJCC stage		
Stage I	175	49.6
Stage II	87	24.6
Stage III	86	24.4
Stage IV	5	1.4
Grade		
G1	55	14.8
G2	180	48.4
G3	124	33.3
G4	13	3.5

**Table 6:** Results of GSEA.

Name	ES	NES	NOM *p* value
GO: positive regulation of osteoclast differentiation	0.70	1.96	0.00*E* + 00
GO: mitochondrial calcium ion transmembrane transport	0.67	1.94	0.00E + 00
GO: acetyl CoA biosynthetic process	0.63	1.81	7.87*E* − 03
GO: very long chain fatty acid metabolic process	0.60	1.78	4.17*E* − 03
GO: pyrimidine nucleoside catabolic process	0.60	1.76	1.89*E* − 03
GO: acetyl CoA biosynthetic process from pyruvate	0.63	1.75	1.58*E* − 02
GO: histone deubiquitination	0.69	1.74	7.74*E* − 03
KEGG: lysine degradation	0.57	1.71	1.62*E* − 02
KEGG: one carbon pool by folate	0.57	1.60	1.41*E* − 02
KEGG: citrate cycle TCA cycle	0.62	1.59	5.26*E* − 02
KEGG: basal transcription factors	0.57	1.58	2.25*E* − 02

Note: ES, enrichment score; NES, normalization enrichment score.

## Data Availability

The datasets used and/or analyzed during the current study are available from the corresponding author upon request.
